# Association between life-course socio-economic status and prevalence of cardio-metabolic risk ractors in five middle-income countries

**DOI:** 10.7189/jogh.08.020405

**Published:** 2018-12

**Authors:** Kemi Ogunsina, Daniel T Dibaba, Tomi Akinyemiju

**Affiliations:** 1 Department of Epidemiology, University of Miami, Miami, Florida, USA; 2 Department of Epidemiology, University of Kentucky, Lexington, Kentucky,USA; 3 Markey Cancer Center, University of Kentucky, Lexington, Kentucky, USA

## Abstract

**Background:**

The burden of non-communicable diseases has increased rapidly in low- and middle-income countries. Past studies have reported an association between socioeconomic status (SES) and cardio-metabolic risk factors, but most have focused on upper income countries. The purpose of this study is to examine the association between SES over the life-course and the burden of cardio-metabolic risk factors in middle-income countries.

**Methods:**

A total of 38 297 adults from China, Mexico, India, South Africa and Russia were included in this cross-sectional study. Life-course SES was defined based on maternal and participant education, and data on blood pressure, body mass index (BMI), self-reported diabetes and hypertension were obtained by trained interviewers. Descriptive, age standardized and multivariable adjusted analyses were conducted using survey weighted statistical procedures in SAS 9.4 (SAS Institute, Cary, NC, USA).

**Results:**

Although 14% of men and 12% of women had current hypertension based on blood pressure measurements, only 2% of men and 4% of women were aware of their hypertensive status. Men with stable high life-course SES had higher odds of being overweight/obese (odds ratio OR = 2.01, 95% confidence interval (CI) = 1.30-3.10), diabetic (OR = 4.82, 95% CI = 2.07-11.2) and hypertensive based on self-report (OR = 3.42, 95% CI = 1.85-6.32) compared to men of low life-course SES. Among women, the odds of being overweight/obese were significantly higher among women with high life-course SES (OR = 1.50, 95% CI = 1.08-2.08).

**Conclusions:**

Higher life-course SES for both men and women was associated with increased odds of overweight/ obesity, and additionally diabetes and hypertension for men in middle income countries.

Cardio-metabolic risk factors such as hypertension, obesity and diabetes are associated with increasing burden of morbidity and mortality globally [1,2]. Chronic diseases associated with these risk factors were responsible for approximately 60% of all deaths globally in 2008 [3,4], an estimate that is projected to increase to 80% by the year 2020[5]. Although the majority of studies evaluating cardio-metabolic risk factors have traditionally focused on upper-income countries, lower- and middle-income country (LMICs) populations are increasingly being identified as highly susceptible due to the epidemiologic transition. The transition is characterized by rapid population growth, ageing, transitions from traditional diets high in fruits and leafy vegetables and high physical activity levels to increased consumption of calorie-dense meals and sedentary lifestyles, leading to increasing rates of obesity [6]. Cardiovascular diseases, cancers, and chronic respiratory diseases account for over two-thirds of deaths in LMICs, and are closely linked with cardio-metabolic risk factors such as diabetes, hypertension and obesity [7].

Socioeconomic status (SES) at both individual [8], parental [9] and across the life-course [8] has been associated with the development of cardio-metabolic risk factors in many upper income countries [10, 11]. In addition, individual and parental SES has been found to predict health behavior and access to preventive health care [12,13]. In high-income countries, lower individual or parental SES has been found to increase the risk of obesity, diabetes and hypertension [10,12,14]; rural residents are also found to have higher risks of cardio-metabolic syndrome compared with urban residents [15]. In LMICs however, fewer studies have examined the association between SES and cardio-metabolic risk factors. SES assessed over the life-course may provide important clues into the accumulation of risk that occurs from childhood, particularly health habits that may predispose individuals to unhealthy diets and low physical activity, leading to increased risk of chronic diseases. Interventions to reduce the burden of chronic diseases in LMICs may benefit from a focus on early life critical periods for both prevention and elimination of cardio-metabolic risk factors. Here, we examine the prevalence of cardio-metabolic risk factors in 5 middle-income countries and their association with life-course SES.

## METHODS

### Data source and study population

The data used for this analysis was obtained from wave one of the World Health Organization (WHO) Study on Global Ageing and Adult Health (SAGE) conducted between 2007 and 2010. The SAGE study was designed as a longitudinal study aimed to assess well-being, quality of life, and access to health care for individuals in developing countries, and obtained data across populations in six different countries; China, Ghana, India, Mexico, South Africa and Russia. A multistage cluster sampling study design was utilized to create nationally representative cohorts from each country. Survey instruments were adapted and modified from the World Health Survey (WHS) and used for individual and household interviews. Trained field staff obtained measurements of blood pressure, height and weight of participants, and a detailed questionnaire translated to the local languages were administered. More information on the SAGE study can be obtained through the WHO SAGE website: (http://www.who.int/healthinfo/sage/cohorts/en/). The current analysis focused on one country from each continent represented in SAGE and included 38 297 participants ages 18 and older from the nationally representative samples of five middle-income countries – China, India, Mexico, Russia and South Africa.

### Cardio-metabolic risk factors

Cardio-metabolic risk factors assessed included body mass index (BMI), current hypertension, history of hypertension, and history of diabetes. BMI was calculated based on measurement of height and weight and categorized into normal weight (BMI 18.5-24.9 kg/m^2^) and overweight/obese (BMI≥25.0 kg/m^2^). Current hypertension was based on the average of 3 blood pressure readings obtained by trained field personnel, and classified as present if average blood pressure reading was ≥140/90 mm Hg. History of hypertension was defined based on self-reported ever diagnosed of hypertension, use of anti-hypertensive medications or diet modification to control hypertension. History of diabetes was defined based on self-reported ever diagnosed of diabetes and the use of anti-diabetes medication or dietary control of diabetes.

### Life-course socio-economic status

Life-course SES was categorized based on maternal and participant educational attainment as follows: stable-high if both mother and participant had ≥ primary school education, increasing if mother had < primary school education while participant had ≥ primary, declining if mother had ≥ primary and participant had < primary school education, and stable-low if both mother and participant had < primary school education. Studies have shown that parental SES, in particular maternal SES, is positively associated with offspring’s overall health [8]. For instance, highly educated mothers have greater decision making powers on the health and welfare of their child, they are more knowledgeable about disease prevention methods [16]. Highly educated mothers are less likely to smoke, or consume excessive alcohol, and more likely to complete prenatal check-ups [17]. These positive early life influences have also been shown to extend to adulthood, with daughters of higher SES mothers less likely to be obese compared with daughters of lower SES mothers[18].

### Covariates

Other covariates included in the analysis were age, gender, marital status, rural/urban residence, country, and current health status. All covariates were analyzed as categorical variables, selected based on the known contribution to the outcome in the presence of other variables.

### Statistical analysis

Survey-weighted frequencies and percentages and χ^2^ tests were used to compare the distribution of baseline demographics and cardio-metabolic risk factors by country. Prevalence of cardio-metabolic risk factors were standardized by age using the world population by age and sex from United States Census Bureau International Data Base [19], using standard age categories: 18-24, 25-39, 40- 64, ≥65years. Survey weighted multivariable logistic regression models were used to determine the association between life-course SES and cardio-metabolic risk factors. All regression models adjusted for age, marital status, rural/urban residence, health status, and country to compute adjusted estimates for the main predictor. For all analyses, *P*-values ≤0.05 were considered statistically significant. All study participants and individual weights were retained in the analysis to ensure valid inferences and representativeness of results to the source population. Although less than 1 percent of study participants had missing data on the main covariates of interest, participants with missing data had higher mean diastolic blood pressure (DBP) (*P* < 0.001), systolic blood pressure (SBP) (*P* < 0.001), and BMI (*P* < 0.001) than participants with complete data but there was no difference in type 2 diabetes status (*P* = 0.763). All statistical analyses were conducted in SAS 9.4 (SAS Institute Inc. NC, USA) using survey weights, strata and cluster variables provided with the SAGE data set, thus enabling study results to be generalizable to the entire population of included countries.

## RESULTS

### Participant characteristics

A total of 38 297 participants were included in this analysis: 16 014 men and 22 283 women ages 18 years and older (**Table 1**); of these, 14 991 participants were from China, 12 198 from India, 2733 from Mexico, 4223 from South Africa and 4152 from Russia. The majority of study participants were married (59%), and more than 45% of both male and female participants in Russia, South Africa, and China had completed a secondary school education; however, there were large gender disparities in completion of secondary school education in India and Mexico. For instance, India had the highest proportion of women with no formal education (55%), and the largest gender disparity in education of 28%. About 84% of men and 82% of women in Russia reported stable high life-course SES, compared with 19% of men and 33% of women in South Africa, 29% of men and 16% of women in Mexico, 20% of men and 19% of women in China, and 12% of both genders in India. Over 50% of men and women in China reported declining SES, while 34% of women and 45% of men in South Africa reported stable low life-course SES.

**Table 1:** Socio-demographic and life-course SES characteristics of SAGE participants

Total (n = 38 297)	China (n = 14 991)		Mexico (n = 2733)		India (n = 12 198)		South Africa (n = 4223)		Russia (n = 4152)	
	**Male (n = 6989)**	**Female (n = 8002)**	**Male (n = 1044)**	**Female (n = 1689)**	**Male (n = 4709)**	**Female (n = 7489)**	**Male (n = 1796)**	**Female (n = 2427)**	**Male (n = 1476)**	**Female (n = 2676)**
**Age group:**										
18 – 24	49 (5.4)	42 (3.3)	5 (6.1)	9 (0.9)	146 (10.2)	964 (18.7)	28 (13.5)	33 (12.1)	24 (15.7)	19 (4.0)
25 – 39	267 (23.1)	371 (27.7)	71 (46.3)	155 (50.6)	515 (34.3)	1961 (36.1)	69 (32.0)	100 (31.9)	76 (28.2)	110 (27.6)
40 – 64	4121 (62.1)	4730 (58.3)	422 (38.2)	704 (38.0)	2612 (46.6)	3357 (36.6)	1118 (47.8)	1390 (46.8)	792 (43.4)	1270 (46.2)
>65	2552 (9.0)	2859 (10.6)	546 (9.3)	821 (10.5)	1436 (8.9)	1225 (8.6)	581 (6.7)	904 (9.2)	584 (12.7)	1277 (22.2)
*P-*value	0.018		0.048		<0.0001		0.896		0.013	
**Marital status:**										
Married	5500 (89.8)	5730 (90.0)	713 (73.0)	803 (66.4)	2436 (86.9)	3297 (80.1)	1325 (70.7)	800 (37.7)	1068 (69.7)	1085 (54.0)
Never married	147 (6.4)	79 (3.2)	60 (22.6)	168 (18.9)	113 (10.1)	239 (7.2)	199 (24.2)	414 (33.5)	49 (18.5)	101 (7.9)
Widow/divorced	517 (3.8)	1343 (6.7)	137 (3.9)	531 (14.6)	208 (2.9)	893 (12.7)	250 (5.1)	1015 (28.9)	215 (11.8)	1188 (38.0)
*P-*value	<0.0001		0.037		<0.0001		<0.0001		<0.0001	
**Highest education:**										
No formal	1786 (14.5)	3384 (24.8)	474 (23.5)	794 (29.4)	983 (27.2)	2666 (54.7)	585 (26.1)	962 (20.7)	14 (0.3)	82 (1.2)
Primary school	1437 (20.7)	1215 (17.1)	210 (20.9)	322 (30.8)	461 (15.6)	667 (16.8)	288 (11.7)	461 (15.4)	85 (2.1)	172 (2.3)
Secondary school	2530 (55.8)	2288 (49.7)	146 (42.4)	200 (29.4)	971 (41.3)	897 (23.6)	299 (56.9)	457 (54.4)	941 (71.6)	1665 (77.1)
College/university	411 (9.0)	265 (8.4)	61 (13.3)	152 (10.4)	342 (16.0)	199 (4.8)	95 (6.3)	82 (9.5)	291 (25.9)	455 (19.4)
*P-*value	0.118		0.325		<0.0001		0.757		0.585	
**Employment status:**										
Unemployed	2474 (17.6)	3400 (27.5)	272 (11.5)	332 (16.2)	735 (10.5)	823 (15.6)	838 (29.9)	1329 (39.7)	719 (23.8)	1539 (35.5)
Private sector	451 (16.6)	242 (9.0)	131 (20.3)	65 (7.6)	199 (9.9)	152 (3.6)	342 (28.5)	212 (11.6)	140 (20.6)	120 (4.2)
Public sector	671 (17.2)	456 (15.5)	54 (10.4)	37 (3.6)	235 (8.5)	95 (2.0)	99 (6.9)	98 (14.9)	408 (42.4)	636 (51.1)
Self-employed	2568 (48.6)	3054 (48.0)	453 (57.9)	1068 (72.6)	1588 (71.1)	3359 (78.6)	345 (34.7)	590 (33.9)	65 (13.2)	79 (4.6)
*P-*value	0.084		0.613		<0.0001		0.046		<0.0001	
**Health status:**										
Good	2515 (56.3)	2415 (49.6)	402 (66.0)	568 (47.8)	1217 (58.8)	1777 (48.5)	763 (71.4)	867 (56.6)	296 (46.0)	304 (37.7)
Moderate	2624 (33.2)	3264 (38.0)	426 (30.0)	756 (41.4)	1211 (33.5)	2082 (41.3)	634 (21.3)	1030 (32.3)	772 (45.5)	1426 (50.0)
Bad	1025 (10.5)	1473 (12.6)	82 (4.0)	178 (10.7)	329 (7.6)	570 (10.2)	227 (7.4)	332 (11.0)	264 (8.4)	644 (12.4)
*P-*valu*e*	0.003		0.056		<0.0001		0.181		0.475	
Life-course SES:*										
Stable high	548 (19.7)	617 (18.2)	96 (28.7)	140 (15.6)	211 (11.6)	475 (12.4)	192 (18.8)	292 (33.4)	835 (83.9)	1483 (81.5)
Increasing	10 (0.2)	26 (0.2)	10 (0.3)	16 (1.5)	7 (0.5)	60 (1.4)	33 (1.4)	42 (1.2)	3 (0.1)	10 (0.1)
Declining	3830 (65.7)	3151 (56.9)	321 (47.2)	534 (54.5)	1563 (61.2)	1288 (32.8)	490 (34.2)	708 (31.4)	482 (15.8)	809 (17.3)
Stable low	1776 (14.3)	3358(24.6)	483 (23.7)	812 (28.4)	976 (26.7)	2606 (53.3)	909 (45.6)	1187 (34.0)	12 (0.3)	72 (1.0)
*P-v*alue	0.066		0.073		<0.0001		0.528		0.023	

SES – socio-economic status, SAGE – Study on Global Ageing and Adult Health

*Based on maternal and participants level of education. *P*-values testing for significant differences between males and females within each country.

### Prevalence of cardio-metabolic risk factors

Mexico had the highest prevalence of overweight/obesity, with age standardized rate of 841 and 699 per 1000 population for men and women respectively, followed by South Africa with prevalence of 676 and 686 per 1000 for men and women respectively (**Table 2**). The lowest rate of overweight/obesity was observed in India, with a prevalence of 134 and 232 per 1000 for men and women, respectively. The age standardized prevalence of current hypertension was highest in South Africa, at 348 and 269 per 1000 for men and women respectively, followed by China, with 168 per 1000 for men. India had the lowest age standardized prevalence of current hypertension, 80 and 84 per 1000 men and women respectively. Across countries, the prevalence of self-reported hypertension was significantly lower compared with current hypertension, except among Chinese men (331 per 1000 self-reported hypertension vs 168 per 1000 current hypertension) and Russian women (182 per 1000 self-reported hypertension vs 149 per 1000 current hypertension). The prevalence of diabetes was highest among Russian women at 132 per 1000 women, followed by Mexican men and women at 84 and 89 per 1000 respectively, and lowest among Russian men (9/1000), Indian women (17/1000) and Chinese men (18/1000). Overall, 13% of the participants had current hypertension but only 24% of them were aware that they had hypertension. Although 14% of men and 12% of women overall had hypertension based on blood pressure measurements, only 2% of men and 4% of women were aware of their hypertension status (data not shown).

**Table 2:** Distribution and age standardized prevalence of cardio-metabolic risk factors per 1000 population

	China		India		Mexico		South Africa		Russia		
	**N (%)**	**Rate**	**N (%)**	**Rate**	**N (%)**	**Rate**	**N (%)**	**Rate**	**N (%)**	**Rate**	
**Overweight/obese:**											
Male	1802 (30.3)	286.2	442 (16.3)	134.0	572 (71.0)	841.4	999 (67.0)	676.0	869 (69.7)		562.4
Female	2627 (37.9)	420.6	1101 (25.2)	232.1	1044 (77.2)	699.4	1555 (77.0)	686.2	1750 (77.6)		477.6
Current hypertension*											
Male	1590 (23.5)	168.2	512 (11.0)	79.6	163 (17.6)	123.7	724 (43.8)	347.7	363 (26.4)		124.9
Female	1674 (21.6)	114.3	590 (8.0)	84.2	215(14.1)	122.0	1014 (46.0)	268.8	818 (32.2)		148.8
**Hypertension – self-reported†**											
Male	1179 (18.1)	330.6	265 (6.2)	44.6	154 (17.5)	61.8	300 (19.1)	55.5	386 (28.4)		119.2
Female	1608 (21.5)	89.1	395 (5.8)	65.0	403 (27.7)	99.8	586 (27.9)	94.2	1178 (47.0)		182.4
**Diabetes:‡**											
Male	299 (4.6)	18.2	153 (3.6)	22.1	116 (13.2)	84.4	103 (6.6)	24.0	47 (3.5)		8.6
Female	398 (5.3)	23.0	131 (1.9)	16.7	239 (16.4)	88.6	178 (8.5)	30.7	194 (7.7)		132.1

*Current hypertension was defined based on mean of three systolic blood pressure ≥140 mm Hg or diastolic blood pressure ≥90 mm Hg.

†Self-reported hypertension was defined as self-reported previously diagnosed hypertension and/or medication use.

‡Diabetes was defined as self-reported diagnosed diabetes on dietary management and/or medication use.

### Life-course SES and cardio-metabolic risk factors

**Figure 1:**
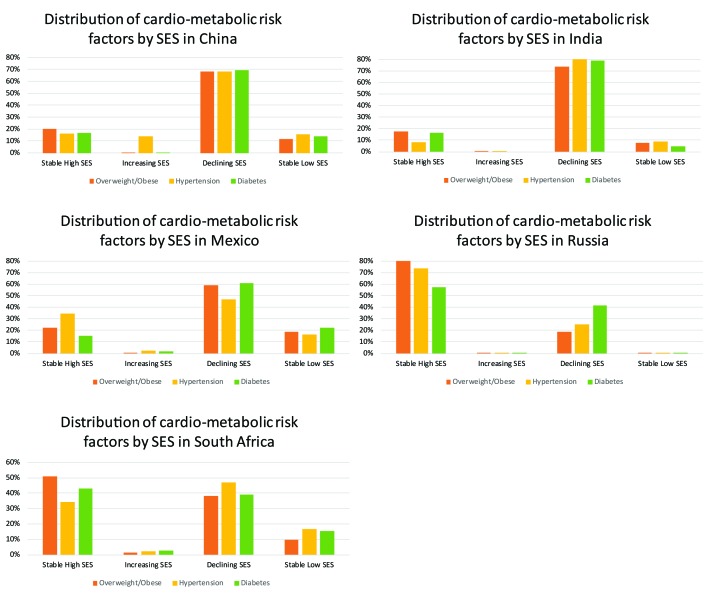
Prevalence of cardio-metabolic risk factors by life-course SES across countries.

Men with stable high (OR = 2.01, 95% confidence interval (CI) = 1.30-3.10) or declining SES (OR = 1.79, 95% CI = 1.34-2.40) had higher odds of overweight/obesity compared with men in stable low SES, while women with stable high (OR = 1.50, 95% CI = 1.08-2.08), increasing (OR = 2.10, 95% CI = 1.10-3.96) or declining (OR = 1.38, 95% CI = 1.18-1.71) life-course SES had higher odds of overweight/obesity compared with women with stable low SES (**Table 3**). In addition, the odds of diabetes were higher among men with stable high (OR = 4.82, 95% CI = 2.07-11.2) and declining (OR = 3.12, 95% CI = 1.93-5.02) life-course SES compared with stable low life-course SES, but there was no significant association among women. There was a 3-fold higher odds of self-reported hypertension associated with stable high compared with stable low life-course SES (OR = 3.42, 95% CI = 1.85-6.32), but this association was only observed among men. Men residing in urban vs rural areas also had increased odds of overweight/obesity (OR = 1.33, 95% CI = 1.03–1.70) as well as diabetes and history of hypertension, although these estimates were not statistically significant. The prevalence of each cardio-metabolic risk factor by life-course SES for each country is presented in **Figure 1**.

**Table 3:** Association between life-course SES and cardio-metabolic risk factors by gender*

	Male n = 12 787				Female n = 17 686			
	**Overweight/obese OR (95% CI)**	**Diabetes OR (95% CI)**	**Self-reported hypertension OR (95% CI)**	**Current hypertension OR (95% CI)**	**Overweight/obese OR (95% CI)**	**Diabetes OR (95% CI)**	**Self-reported hypertension OR (95% CI)**	**Current hypertension OR (95%CI)**
**Life-course SES:†**								
Stable high	**2.01 (1.30-3.10)**	**4.82 (2.07-11.2)**	**3.42 (1.85-6.32)**	1.17 (0.72-1.92)	**1.50 (1.08-2.08)**	0.81 (0.34-1.91)	0.83 (0.54-1.27)	0.78 (0.52-1.18)
Increasing	0.96 (0.34-2.68)	1.57 (0.28-8.78)	0.90 (0.23-3.64)	0.42 (0.15-1.18)	**2.10 (1.10-3.96)**	0.85 (0.30-2.43)	0.62 (0.34-1.13)	1.35 (0.55-3.34)
Declining	**1.79 (1.34-2.40)**	**3.12 (1.93-5.02)**	1.33 (0.99-1.81)	0.98 (0.71-1.35)	**1.38 (1.18-1.71)**	1.00 (0.59-1.70)	0.99 (0.75-1.33)	0.92 (0.71-1.19)
Stable low	Ref	Ref	Ref	Ref	Ref	Ref	Ref	Ref
**Age**								
18–24	0.51 (0.21-1.28)	**0.09 (0.03-0.29)**	**0.01 (0.00-0.03)**	**0.20 (0.08-0.50)**	**0.23 (0.14-0.37)**	**-**	**0.01 (0.00-0.06)**	**0.09 (0.03-0.25)**
25–39	**0.64 (0.45-0.90)**	**0.06 (0.02-0.18)**	**0.04 (0.02-0.10)**	**0.28 (0.19-0.40)**	**0.31 (0.22-0.44)**	**0.06 (0.03-0.16)**	**0.02 (0.01-0.04)**	**0.14 (0.10-0.21)**
40–64	1.01 (0.82-1.25)	**0.30 (0.21-0.42)**	**0.21 (0.16-0.28)**	**0.63 (0.51-0.78)**	**0.73 (0.59-0.89)**	**0.42 (0.27-0.68)**	**0.26 (0.21-0.32)**	**0.51 (0.41-0.62)**
≥65	Ref	Ref	Ref	Ref	Ref	Ref	Ref	Ref
**Marital status:**								
Married	1.94 (0.94-4.00)	1.84 (0.69-4.93)	1.73 (0.66-4.51)	1.46 (0.83-2.57)	**2.00 (1.19-3.31)**	1.23 (0.63-2.40)	0.99 (0.52-1.88)	1.02 (0.58-1.80)
Wid/divorced	1.50 (0.62-3.63)	2.02 (0.68-6.06)	1.97 (0.54-7.18)	1.15 (0.63-2.13)	1.21 (0.68-2.17)	1.46 (0.74-2.88)	0.77 (0.41-1.45)	1.12 (0.62-2.05)
Never married	Ref	Ref	Ref	Ref	Ref	Ref	Ref	Ref
**Residence:**								
Urban	**1.33 (1.03-1.70)**	1.62 (0.96-2.76)	1.49 (0.93-2.36)	0.91 (0.68-1.23)	0.86 (0.71-1.04)	1.60 (0.99-2.59)	1.28 (0.95-1.74)	**0.63 (0.48-0.82)**
Rural	Ref	Ref	Ref	Ref	Ref	Ref	Ref	Ref
**Country:**								
China	**0.23 (0.14-0.39)**	0.74 (0.35-1.57)	1.08 (0.68-1.70)	**0.32 (0.19-0.55)**	**0.13 (0.08-1.04)**	0.78 (0.48-1.27)	**0.65 (0.43-0.98)**	**0.30 (0.21-0.44)**
India	**0.11 (0.06-0.18)**	1.13 (0.48-2.67)	0.74 (0.44-1.23)	**0.14 (0.08-0.25)**	**0.10 (0.06-0.17)**	0.63 (0.38-1.04)	**0.44 (0.29-0.67)**	**0.17 (0.12-0.26)**
Mexico	**3.09 (1.45-6.58)**	**4.52 (1.65-12.4)**	0.71 (0.40-1.27)	**0.26 (0.13-0.52)**	1.08 (0.57-2.06)	**3.30 (1.81-6.02)**	0.74 (0.46-1.18)	**0.24 (0.14-0.41)**
Russia	0.64 (0.31-1.34)	**0.20 (0.08-0.49)**	1.10 (0.58-2.12)	**0.24 (0.13-0.47)**	**0.39 (0.20-0.77)**	0.92 (0.44-1.91)	**2.10 (1.34-3.29)**	**0.56 (0.36-0.89)**
South Africa	Ref	Ref	Ref	Ref	Ref	Ref	Ref	Ref

SES – socio-economic status, OR – odds ratio, CI – confidence interval

*OR and 95% CI reported adjusted for age, marital status, country, rural/urban residence, health status and socioeconomic status. Statistically significant ORs are bolded.

†Life-course SES variable was based on maternal and participant level of education.

## DISCUSSION

In a nationally representative sample of adults in five middle-income countries (China, South Africa, India, Russia and Mexico), we observed that the population distribution of stable high life-course SES ranged from 18% in China to over 80% in Russia. In addition, the highest age-standardized rates of overweight/obesity, history of hypertension, and diabetes were observed in Mexico, South Africa, Russia, respectively. Across the included countries, men with stable high life-course SES had two to four times higher odds of being overweight/obese, diabetic and hypertensive compared with men of stable low life-course SES. While women with stable high, declining and increasing life-course SES had up to a 2-fold increased odds of overweight/obesity compared with stable low life-course SES. We also observed that a significant proportion (13%) of participants were hypertensive based on blood pressure measurements obtained during in-person interviews, but 76% them had no prior knowledge of having hypertension. The largest disparity between self-reported history of hypertension and current hypertension was observed in South Africa where only 6% of men with hypertension were aware that they had hypertension or high blood pressure, whereas 38% had hypertension based on blood pressure measures.

The associations observed in this study are consistent with existing studies showing that in lower and middle income countries, higher SES individuals have a higher prevalence of obesity, diabetes and hypertension compared with lower SES individuals, while in high-income countries, the association is typically inverse [20-22]. Few studies have evaluated SES over the life-course in lower and middle-income countries, an area that deserves greater attention given the rapid economic development and effects of globalization in these countries. Life-course SES is hypothesized to influence adult health through the accumulation of different SES experiences throughout life; increasing life-course SES indicates that higher adult SES may attenuate the negative effects of early life low SES, while decreasing life-course SES indicates that lower adult SES may have a negative influence on adult health despite relatively higher SES in early life [23]. Low SES across the life-course is associated with increased prevalence of obesity, diabetes and hypertension [18] in developed countries; however, results on the influence of increasing and declining life-course SES have been mixed. In the countries evaluated in this study, the association between life-course SES and cardio-metabolic risk factors was most consistent for overweight/obesity among both men and women, with significantly higher odds for those with stable high, declining or increasing life-course SES. History of diabetes and hypertension were also significantly higher among men with stable high life-course SES, but not among women. Other studies from developing countries have reported conflicting results; some studies report a direct or no association between SES and cardio-metabolic risk factors [24-27], while other studies observed that the association varies by geographic location [28], or on the measures of SES used [29]. These differences may be due to between country heterogeneity in the level of economic development, ie some middle-income countries are likely more economically developed than others, eg despite both being classified as middle-income countries, almost 20% of women in Russia had completed college/university education, compared with 5% in India. It may also be due to within country differences in the association between SES and lifestyle based on social norms such as cultural standards of ideal body weight.

The potential mechanisms explaining the association between SES and cardio-metabolic risk factors in the studied countries may include socially patterned behavioral risk factors such as alcohol use, dietary quality and physical activity [20]. Alcohol consumption of greater than 7 drinks per week has been associated with obesity especially in men [30], and increased alcohol consumption is associated with higher income. Dietary patterns in many middle-income countries have also changed dramatically as a result of economic development and globalization.The concentration of overweight/obesity among higher SES groups suggest that lack of affordability of the relatively new dietary items may act as a protective factor against increasing obesity rates among lower SES groups. A third potential mechanism involves access to health care- higher life-course SES was associated with increased odds of self-reported hypertension, but not interviewer measured hypertension based on high blood pressure. In addition, only a minority of adults who were hypertensive based on blood pressure measurements were aware of their hypertensive status. This is likely due to improved access to health care among higher SES groups, leading to clinical diagnosis, and potentially treatment, for hypertension, and under-estimation of hypertension prevalence in lower SES groups [31-33]. A separate study in China also observed that only 28% of diabetic patients were aware of their diabetic status, and only 23% had received treatment [26]. Studies in the US show that higher SES individuals are often the first to adopt health measures to enhance their health and overall well-being by taking advantage of known protective health factors, thus explaining the SES gradient observed in health [34].

Cardio-metabolic risk factors are implicated in the etiology of multiple non-communicable diseases such as cardiovascular diseases and cancer, with increasing prevalence of those conditions in LMICs, and accounting for increasing majority of deaths worldwide [3]. Findings of higher prevalence of overweight/obesity, diabetes and hypertension among high-income individuals in this study highlights a critical disconnect between socio-economic resources and utilization of health care. The behavioral factors associated with cardio-metabolic risk factors eg, sedentary lifestyle, poor diet, excessive alcohol consumption, tend to increase with increasing SES, however our results suggest that higher utilization of medical care for diagnosis and treatment typically observed among high SES individuals has not yet resulted in improved cardio-metabolic health in these populations. In addition, while rural residents may be less likely to have cardio-metabolic risk factors due to healthier dietary and physical activity patterns, lack of access to care is likely more challenging compared with urban areas. Country- and region- specific interventions focused on increasing awareness of chronic diseases risk factors, promoting healthy dietary and physical activity lifestyles, and highlighting the importance of preventive health care will be important in reducing the higher burden of non-communicable diseases (NCDs) among high SES groups [35-37]. Our observation of significantly higher odds of overweight/obesity, diabetes and self-reported hypertension among men with stable-high and declining life-course SES also suggests that these interventions should begin in early life- as this may be a critical period during which adverse and protective health behaviors are developed and then sustained over the life-course. For women, the stronger associations observed with increasing life-course SES also suggests that health behaviors likely accumulate over the life-course, and intervention strategies in early life, adolescence and adulthood will likely be needed. Across these countries, concerted efforts to improve the health care delivery systems will be needed to meet future health challenges [37].

### Strengths

This study evaluated data on a large sample of adult representative of five middle-income countries, therefore enhancing the generalizability of these results. In-person interviews and biometric measures were obtained directly by well-trained WHO field staff, enhancing the quality and internal validity of our study variables. Life-course SES was measured consistently with other studies, and the current analysis focused on maternal and individual education since these variables have been observed to be highly correlated with health behaviors in prior studies [38].

### Limitations

Data on SES and certain risk factors were based on self-reported information which may be vulnerable to recall bias. Classification of participants on the risk factor measurements might underestimate the number of those with the risk factors or participants with ongoing treatment, which may bias the result towards the null. In addition, we used BMI to assess overweight/obesity although studies suggest that central obesity may be a more important measure of adiposity in certain population groups.

## CONCLUSIONS

Stable high and declining life-course SES was associated with increased odds of overweight/obesity, diabetes and hypertension among men in the middle-income countries examined. Among women, stable high, increasing and declining life-course SES was also associated with increased odds of overweight/obesity. These patterns suggest that the influence of SES on adult health for men may operate during critical periods in early life, while for women, it may be accumulative over the life-course. The majority of adults who were hypertensive based on blood pressure measurements by trained interviewers were unaware of their condition, indicating an urgent need for increased awareness, primary prevention and treatment.
